# Pigmented Conjunctival Nevus: A Mystery Solved by Histopathology

**DOI:** 10.7759/cureus.64528

**Published:** 2024-07-14

**Authors:** Charusheela Gore, Mangesh Londhe, Sushama Gurwale, Pranjali Nibe

**Affiliations:** 1 Pathology, Dr. D.Y. Patil Medical College, Hospital and Research Centre, Pune, IND

**Keywords:** benign lesion, pediatric, melanomas, inflammatory juvenile conjunctival nevus, conjunctival lesion

## Abstract

Inflammatory juvenile conjunctival nevi (IJCN) is a rare type of nevus and its clinical presentation overlaps with that of malignant conjunctival melanoma. It is a benign lesion that has been described to progress to melanoma in some cases. IJCN may clinically mimic melanoma due to its rapid growth features and atypical histology. Thus, its accurate diagnosis by histopathology is a prerequisite for proper management. Here, we present a case of conjunctival lesion mimicking melanoma clinically.

## Introduction

Conjunctival lesions represent only 2.5% of ophthalmological pathology and more than 50% are of melanocytic origin [1]. Out of all the pigmented conjunctival lesions that are removed, 53% are melanocytic lesions. Among these lesions, nevi are the most common (52%), followed by melanoma (25%), primary acquired melanosis (PAM) in 21%, and racial melanosis in 3% [2]. Among nevi, inflammatory juvenile conjunctival nevi (IJCN) are amelanotic and often benign lesions located near the limbus. They typically appear throughout adolescence and early adulthood [3]. IJCN may clinically mimic melanoma attributed to its features of rapid growth and atypical histology. Most IJCN cases can be treated with antiallergics and observation; however, excision of the lesion is indicated if the malignancy is suspected. In such cases, its accurate diagnosis by histopathology is essential because if it's melanoma, then the treatment is early excision with negative margins to avoid disease spread, recurrence, or metastasis. Here, we present an eight-year-old female child with recent rapid growth of conjunctival lesion mimicking melanoma clinically.

## Case presentation

**Figure 1:**
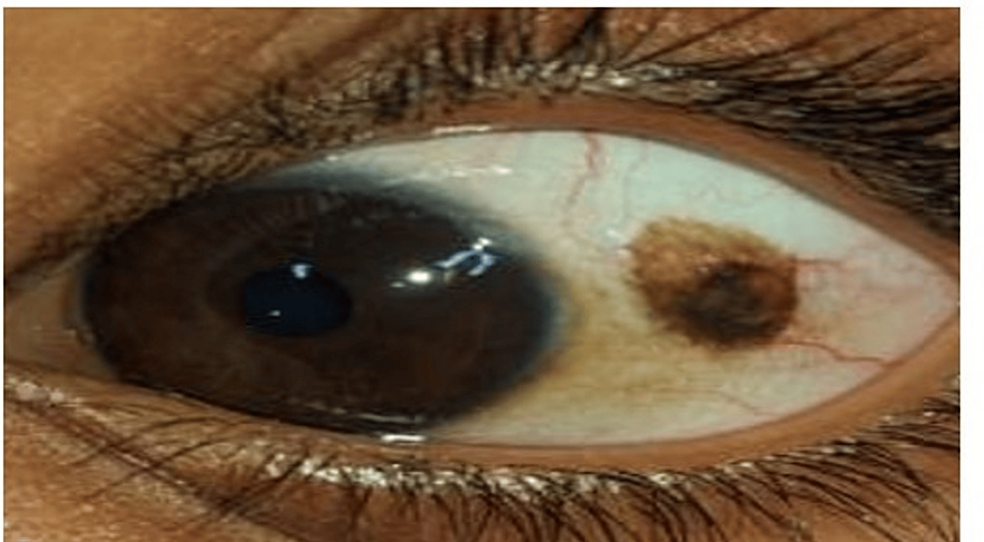
Clinical image showing heterogeneously hyperpigmented lesion along with dilated blood vessels The figure shows heterogeneously hyperpigmented lesion located 3 mm from the limbus temporally along with dilated blood vessels present at 2 o'clock, 5 o'clock and 10 o'clock.

An eight-year-old girl came along with her mother who was complaining of a brown-coloured lesion in the left eye of the child, which was present since her birth. This lesion was painless and nonprogressive until one month before presenting, during which it showed a rapid increase in size. The patient also gave a history of itching, foreign body sensation, and heaviness of eyelids in both eyes for two months. There was no history of diminution of vision, double vision, redness or discharge, ocular trauma or surgery. There was no significant past or family history. General and systemic examination was within normal limits. Ocular examination revealed distance vision of 6/6 and N6 for near vision in both eyes. Extraocular movements were full, free, and painless. Intra ocular pressure was 14 mmHg which was within normal limits. An anterior segment examination showed papillae over the upper palpebral conjunctiva in both eyes, whereas the left eye revealed a single, elevated, oval, heterogeneously hyperpigmented cystic interpalpebral bulbar lesion (5x3 mm) with well-defined margins located 3 mm from the limbus temporally along with dilated blood vessels present at 2 o'clock, 5 o'clock, and 10 o'clock (Figure 1).

The rest of the parameters were within normal limits along with the fundal examination. Slit-lamp biomicroscopy was done, and it confirmed a raised pigmented lesion in the nasal limbal conjunctiva along with edema in the left eye. The rapid growth of the lesion raised a clinical suspicion of conjunctival melanoma. The patient was posted for conjunctival mass excisional biopsy with a 5 mm clear margin and and no touch technique with amniotic membrane grafting under general anesthesia. The excised lesion was submitted for histopathological examination (HPE).

**Figure 2:**
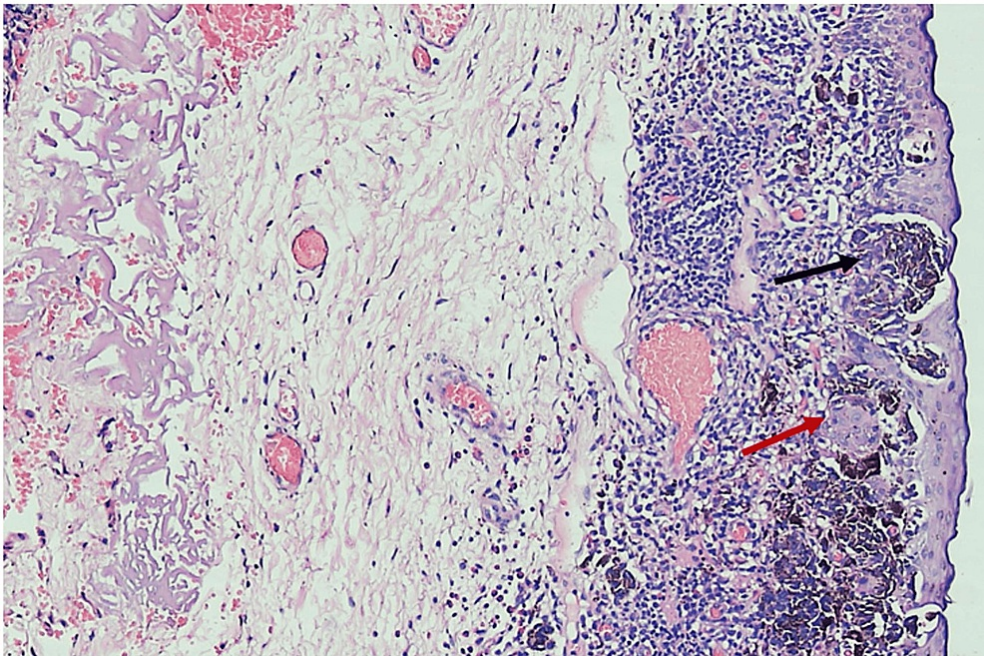
Photomicrograph shows nests and islands composed of type A (melanin containing) and type B (non-melanin containing) nevus cells surrounded by inflammatory cells. Type A (melanin containing - black arrow) and type B (non-melanin containing - red arrow)

Grossly received a single, grey-brown soft tissue piece of size 1 X 0.8 X 0.5 cm, that was formalin-fixed, and all the tissue was processed for further routine examination. On microscopy, hematoxylin and eosin stained sections studied showed stratified squamous epithelium with loose collagenous subepithelial stroma. The junction of these two revealed nests and islands composed of type A (melanin containing) and type B (non-melanin containing) nevus cells. These nevus cells were surrounded by inflammatory cells consisting of lymphocytes, plasma cells, and eosinophils (Figure 2).

**Figure 3:**
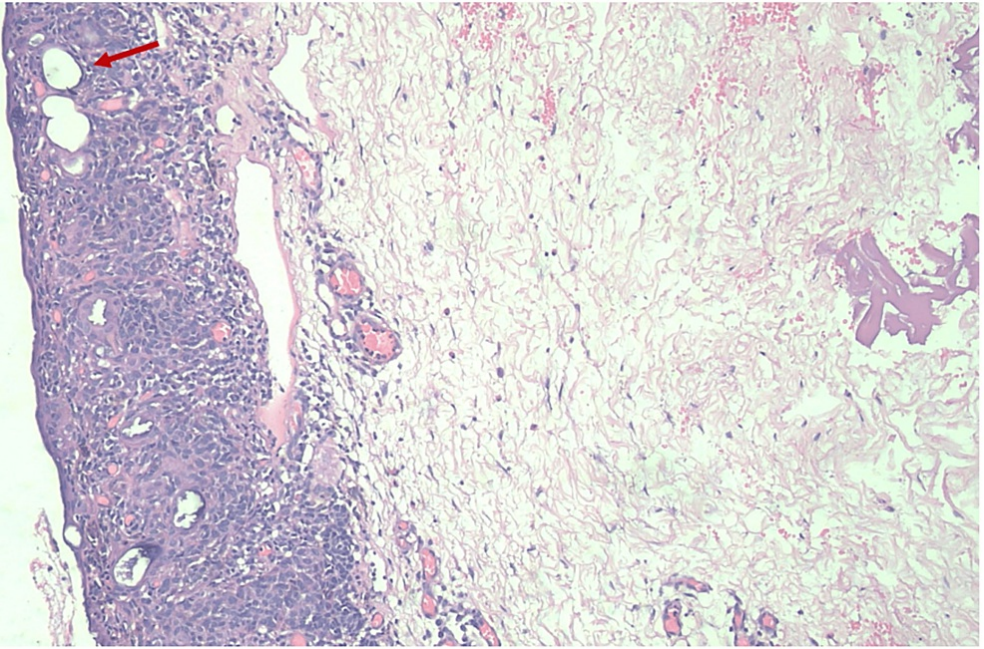
Photomicrograph showing few epithelial cysts amidst the nevus cells within the epidermis (red arrow)

Few epithelial cysts were noted amidst the nevus cells within the epidermis (Figure 3). 

There was no evidence of increased or atypical mitosis, or eosinophilic macronucleoli or epithelial dysplasia, or malignancy. Based on these microscopy findings conjunctival melanoma was ruled out and a final diagnosis of IJCN was made.

## Discussion

Conjunctival tumors may be benign or malignant. A retrospective analysis of 806 cases of conjunctival tumors in young adults (less than 21 years old) by Shields et al. revealed that 97% of the cases were benign and 3% were malignant [4]. IJCN are benign lesions but have been described to progress to melanoma in 1% of cases and present clinically in the first or second decade of life [1]. A nevus begins as a small nest of cells, and the juxtralimbal conjunctiva is the most commonly reported site of IJCN. It is associated with allergic conjunctivitis, vernal conjunctivitis, and systemic allergies in few cases [5].

A rapid increase in the size of the lesion together with an increase in pigmentation raises the suspicion of malignancy. In our case, the patient had no other complications such as allergic conjunctivitis or systemic allergies. The lesion was present from birth but rapidly enlarging for only one month before presenting to an outpatient department, and a provisional clinical diagnosis of malignant conjunctival melanoma was therefore made.

Histopathologically, it may resemble other lesions, including conjunctival cyst, inflamed pinguecula, episcleritis, foreign body granuloma, allergic conjunctivitis, lymphangioma, squamous neoplasia, conjunctival sarcoidosis, and leukemia [6]. IJCN has similar presentations that overlap with malignant conjunctival melanoma, which leads to great difficulty in its clinical differentiation from conjunctival melanoma and makes it the closest and essential clinical as well as histopathological differential diagnosis. Since most patients with IJCN are in the younger age group, which makes resection of the tumor under local anesthesia difficult. The recurrence rate after simple excision of IJCN is reported to be 12% [7].

IJCN and melanoma, although they have overlapping clinical as well as histological features, it is very important to make an accurate diagnosis of pigmented tumor, and therefore resection and HPE play a vital role. Clinical signs suggestive of malignancy include the rapid growth of a lesion, presence of feeder vessels, irregularities of the margins, and color changes. In such cases, excision of the lesion is mandatory [8,9]. In our case, the rapid growth along with the presence of feeding vessels clinically indicated the lesion as melanoma. If malignant transformation occurs, there is a significant mortality (13% in 10 years). Therefore, rapid diagnosis and treatment is essential. Majority of IJCN can be treated with antiallergics and observation; however, excision of the lesion is indicated if the malignancy is suspected. Whereas, excisional resection of the lesion remains the mainstay of treatment in the cases of malignant melanoma.

## Conclusions

As IJCN and malignant melanoma mimic each other clinically, it is necessary to distinguish them and establish the correct line of treatment. Clinical examination and HPE play a vital role in establishing an accurate diagnosis in such cases. In pediatric patients with IJCN, observation with close follow up can be helpful, thus avoiding any untoward surgery related complications.
